# Memory B cell and antibody responses to flavivirus infection and vaccination

**DOI:** 10.12703/r/10-5

**Published:** 2021-01-25

**Authors:** Awadalkareem Adam, Servando Cuellar, Tian Wang

**Affiliations:** 1 Department of Microbiology & Immunology, University of Texas Medical Branch, Galveston, TX, 77555, USA; 2 School of Medicine, University of Texas Medical Branch, Galveston, TX, 77555, USA; 3 Department of Pathology, University of Texas Medical Branch, Galveston, TX, 77555, USA; 4 Sealy Institute for Vaccine Sciences, University of Texas Medical Branch, Galveston, TX, 77555, USA

## Abstract

Flaviviruses are a group of mosquito- or tick-borne single-stranded RNA viruses that can cause a wide range of clinical manifestations in humans and animals, including asymptomatic, flu-like febrile illness, hemorrhagic fever, encephalitis, birth defects, and death. Many of them have no licensed vaccines available for human use. Memory B cell development and induction of neutralizing antibody responses, which are important for the control of flavivirus infection and dissemination, have been used as biomarkers for vaccine efficacy. In this review, we will discuss recent findings on memory B cells and antibody responses from studies in clinical specimen and animal models of flavivirus infection and vaccination with a focus on several clinically important flaviviruses, including dengue, West Nile, yellow fever, and Zika viruses.

## Introduction

Flaviviruses are a group of single-stranded RNA viruses that are primarily transmitted by ticks or mosquitoes. Among them, there are several medically important human pathogens, such as dengue virus (DENV), yellow fever virus (YFV), West Nile virus (WNV), and Zika virus (ZIKV)^1,2^. Flaviviruses cause a wide range of clinical manifestations in humans and animals ranging from asymptomatic, self-limiting flu-like febrile illness to hemorrhagic fever, encephalitis, birth defects, and death^2^. No antiviral therapeutics are currently available. The flaviviral genome encodes three structural proteins (capsid, pre-membrane [PrM], and envelope [E]) and seven nonstructural proteins (NS1, NS2A, NS2B, NS3, NS4A, NS4B, and NS5)^3–5^. The E protein has been reported to be involved in viral entry into host cells and is, thus, an important target for the induction of B cell and neutralizing antibodies (NAbs)^6^. The protein can be further divided into three domains: EDI, EDII, and EDIII. In addition, the NS1 and PrM proteins can serve as dominant targets for the human B cell response against flaviviruses^7–9^.

Vaccine development has been successful in the control of several flaviviruses, such as Japanese encephalitis virus (JEV), tick-borne encephalitis virus (TBEV), and YFV. Humoral immunity, which comprises B cell and antibody responses, plays an important role in host protection against flavivirus infection^10,11^. In particular, the development of memory B cells (MBCs) and induction of NAb responses are critical for the control of viral infection and dissemination and, thus, are important biomarkers for vaccine efficacy^12^. Here, we mainly focus on discussion of recent progress in understanding the role of MBCs and antibody responses against flavivirus infection and vaccination.

## Memory B cells

B cells are lymphocytes generated in the bone marrow from lymphoid precursors via a process involving the recombination of V, D, and J gene segments coding for the variable region of the immunoglobulin (Ig) heavy and light chains^13^. Mature naïve B cells express B cell receptor (BCR) such as IgM and IgD molecules. Following viral infection or vaccination, antigen stimulation of B cells through the BCR triggers the activation of naïve B cells within a few days at the T cell–B cell follicle border to eventually form follicular germinal centers (GCs), which generate long-lived plasma cells (LLPCs) producing IgG NAbs and antigen-specific MBCs within 7 days^14,15^. In extrafollicular foci, antigen-activated B cells can differentiate into short-lived antibody secreting cells (ASCs)^16,17^. MBCs generated in the GC during the primary immune response circulate at low frequencies throughout the body as resting lymphocytes, which may persist for decades^18^. Upon antigen re-exposure, MBCs are activated, proliferate quickly (within 2 to 3 days) and more robustly than naive B cells, and differentiate into high-affinity IgG ASCs^13,18^. This activation also generates new antigen-specific LLPCs and MBCs. During primary flaviviral infection, there is a rapid and transient increase in antibody-secreting plasmablasts. At the convalescent stage, MBCs and LLPCs both contribute to long-term humoral immunity. Upon secondary flavivirus infection, MBCs are mostly characterized as highly cross-reactive to other genetically related flaviviruses.

## MBC and antibody responses to flavivirus infection and vaccination

### DENV

DENV infection is the most prevalent flavivirus infection, with about 390 million human cases annually in the tropical and subtropical regions worldwide^19^. The WHO has estimated that 50% of the world’s population is at risk of DENV transmission. Based on antigenic determinants or nucleotide sequences of DENV E, Pre-M, or NS1 protein, there are four serotypes of DENV, namely DENV1, DENV2, DENV3, and DENV4^20^.

**Figure 1.**
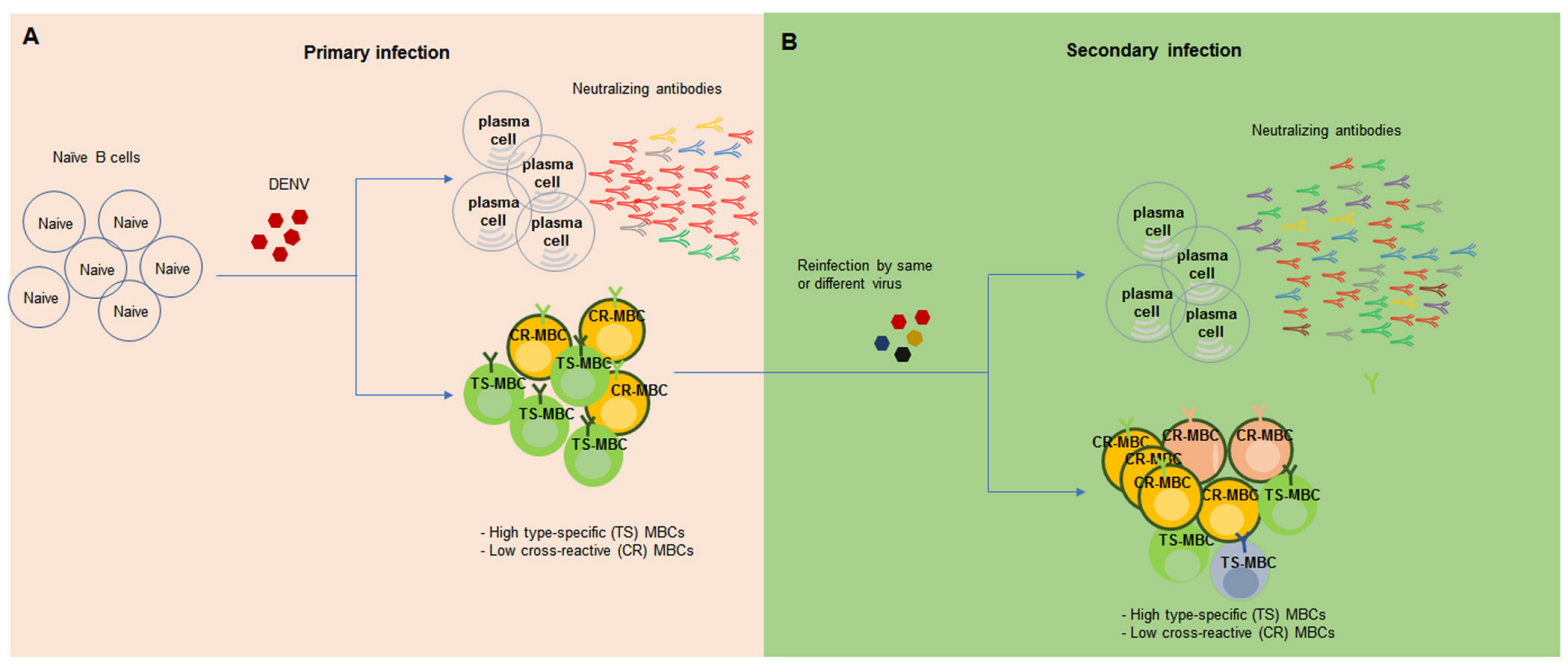
B cell responses following primary and secondary dengue virus (DENV) infections. **A**: During primary DENV infection, virus-specific naïve B cells are activated and differentiate into antibody-secreting long-lived plasma cells (LLPCs) and memory B cells (MBCs). **B**: During secondary infection with a different serotype DENV, MBCs are activated, produce cross-reactive antibodies with high avidity, and are expanded. New cross-reactive MBCs and LLPCs are also generated.

Following a cutaneous DENV infection in immunocompetent mice (mimicking a mosquito bite), there was massive early activation and strong proliferation of B cells, but poor or almost absent T cell responses, which suggest a major role for humoral immunity during DENV infection^21^. One early study showed that the cross-reactive antibodies produced by both LLPCs and MBCs provided cross protection against sequential heterotypic DENV infection in AG129 mice (IFN-α/β and IFN-γ receptor deficient). Nevertheless, most of the MBC studies to date have been conducted in human samples because of the limitation of physiologically relevant DENV infection animal models^22^. The predominant B cell responses during acute primary infection in humans are the CD27- and CD38-expressing DENV-specific plasmablasts, which produce DENV serotype-specific antibodies that mainly target the quaternary structure epitopes centered on DENV EDIII. The secretion of soluble CD27 and CD38 in the plasma was reported to be associated with the activation of the plasmablast during acute DENV infection^23^. B cells specific for DENV E, Pre-M, and NS1 were detectable in patients with primary DENV infection. In particular, DENV E-specific B cells had the highest frequencies and were highly serotype specific. However, during secondary DENV infection, DENV E-specific B cells were mostly serotype cross-reactive, which produced high-avidity antibodies^23,24^ (Figure 1A). Analysis of the diversity and antigen specificity of the plasmablast antibody repertoire elicited during primary DENV infections revealed that a high proportion of the DENV-elicited plasmablasts express IgA, in addition to IgG and IgM class-switched cells. Furthermore, these IgA class-switched cells were extensively hypermutated^25^. Upon secondary DENV infection, small subsets of MBCs become activated as plasmablasts^26^. Single-cell analysis of B cells suggests that similar MBC responses were induced during primary and secondary DENV infection. However, during secondary infection, MBC responses were more cross-reactivated to other closely related flaviviruses, such as ZIKV^27^. The E-specific B cells in patients were serotype cross-reactive and secreted antibodies with higher avidity to heterologous DENV serotypes during secondary infection^24,28,29^. Characterization of serum samples from the secondary DENV infection reveals cross-reactive antibodies to all DENV serotypes (Figure 1B)^30^. Furthermore, plasmablast-derived monoclonal antibodies (mAbs) in DENV exposure donors were broadly cross-reactive against DENV 1–4, displayed poorly neutralizing activity, and showed antibody-dependent enhancement (ADE) effects *in vitro*^31^

Screening of samples from a DENV-endemic area suggested that more than 80% of subjects developed MBCs but the frequencies of these cells varied significantly^32^. The cross-reactive MBCs expanded during the first years of DENV infection and were retained in some donors for 3 to 4 years after initial infection. The antigenic specificity towards DENV serotypes or more distantly related flaviviruses, including ZIKV, also varied. Some donors had strong MBC responses to all four DENV serotypes that persisted for up to 5 years^33^. In addition, MBC-derived antibodies were shown to bind to E, PrM, and NS1, which is more in line with the specificity of serum antibodies produced following re-infection and during the early convalescence phase^26^. Despite their low frequency in peripheral blood, human mAbs derived from purified MBCs have been characterized by several groups^34–36^. For example, mapping of human MBCs and serum NAb responses to DENV serotype 4 infection and vaccination revealed that antibodies derived from MBCs and LLPCs bind to the quaternary structure epitopes close to the hinge region between EDI and EDII^34^. Young *et al*. generated 15 DENV3 type-specific mAbs from MBCs isolated from children naturally infected with DENV and demonstrated that these antibodies recognize E glycoprotein^37^. Multiple approaches have also been developed for the assessment of the breadth and the durability of DENV/ZIKV B cell responses at the single-cell level following natural infection or vaccination. One group utilized the Alexa Fluor dye-labeled DENV to identify a small frequency of B cells from DENV immune individuals by flow cytometry analysis^38^. Others developed DENV/ZIKV FluoroSpot assays for the detection and enumeration of DENV-specific, ZIKV-specific, and DENV/ZIKV cross-reactive MBCs^39,40^. These assays are sensitive tools to detect the specific and cross-reactive MBCs.****

The generation of broadly NAbs (bNAbs) by B cells against all four DENV serotypes is crucial for DENV vaccine development. Durham *et al*. previously used transcriptomic analysis and identified novel recognition determinants of bNAbs within the DENV EDI region^41^.

Vaccination with TAK-003, a live attenuated tetravalent DENV vaccine candidate, which includes both attenuated DENV2 and three chimeric viruses containing the PrM/E of DENV1, 3, and 4 on the DENV2 backbone, induces both type-specific and cross-reactive MBCs to all four DENV serotypes. Thus, unlike natural DENV infection, all four components of TAK-003 contribute to the DENV-specific MBC response following vaccination.

### WNV

WNV infection in humans induces West Nile fever and neuroinvasive diseases, including meningitis, encephalitis, acute flaccid paralysis, and death^42^. In addition, WNV convalescent patients developed long-term neurological sequelae or chronic kidney diseases, or both^43–51^.

Mature B cells play an important role in host protection against WNV infection. Mice with genetic deficiency in B cells and antibody were much more susceptible to lethal WNV infection^10^. Furthermore, evidence suggests that immature B cells also provide host protection against WNV infection. For example, immunization of B cell-activating factor receptor (BAFFR)^–/–^ mice, which have normal levels of immature B cells but reduced numbers of mature B cells, protects against lethal WNV infection^52^.

The persistence of WNV-specific MBCs and ASCs in WNV convalescent patients was first reported by Tsioris *et al*.^53^. In addition, they identified four novel WNV NAbs using the single-cell analysis method and NGS analysis. Vaccination of mice with the inactivated cell culture JEV antigen in the presence of Advax delta inulin adjuvant (JE-ADVAX) also triggered MBC responses and serum cross-NAb production, which together provided heterologous protection against WNV challenge^54^. Furthermore, while both MBCs and LLPCs persist long after WNV clearance, depletion and adoptive transfer studies demonstrate that MBCs can respond to variant viruses that escape NAb produced by LLPCs without acquiring additional somatic mutations. In contrast, LLPC function was limited to neutralizing homologous viruses upon reinfection^55^.

The underlying mechanisms of MBC development during WNV infection are not well understood. It was reported that the expression of myeloid differentiation primary response 88 (MyD88) is required for B cell activation, development of GCs, and generation of LLPCs and MBCs following immunization with RepliVAX WN, a single-cycle flavivirus vaccine candidate derived from WNV. In contrast, the expression of Toll-like receptor (TLR) 3, which is independent of MyD88, is important for the maintenance of GCs and development of LLPCs but not for the differentiation of MBCs^56^. Thus, pathogen recognition receptor (PRR)-mediated innate immune signaling pathways could play differential roles in MBC and antibody responses upon WNV vaccination.

### YFV

Yellow fever caused by YFV is endemic in South America and Africa. It is characterized by fever, vomiting, nausea, hepatitis with jaundice, renal failure, hemorrhage, and death^57^. YFV 17D is a highly live attenuated vaccine that was developed by Max Theiler in the 1930s. The three 17D substrains (17D-204, 17DD, and 17D-213) have minor differences in genome sequences, but all have proved to be effective vaccines^58^.

Mouse model studies suggest that the vaccine induces long-lasting humoral responses and provides protection along with memory CD4^+^ T cell immunity^59^. Wec *et al*. recently characterized MBC responses following YFV17D vaccination by using a high-throughput single B cell cloning technology. Early MBC responses were mediated by both the classical immunoglobulin M (IgM^+^CD27^+^) and the switched immunoglobulin (swIg^+^) MBCs. The swIg^+^ MBC populations and atypical IgM^+^ and IgD^+^ MBCs were stable over time. However, the classical IgM^+^CD27^+^ MBCs declined quickly. In addition, the NAb response was found to target a fusion loop-proximal antigenic site within the YFV EDII protein^60^.

Vaccination of healthy donors with the live attenuated vaccine YFV 17D triggers the activation of innate and adaptive immune responses. Induction of strong YFV-specific neutralization titers are correlated with soluble IL-6R levels and activation of CD4^+^ T cell responses during the early stage of vaccination^61^. Immune activation promotes strong NAb titers. However, another study pinpointed that the pre-active environment in vaccinees, such as the presence of proinflammatory monocytes and activation of CD8^+^ T cells and B cells, contributes to lower neutralization titers and reduction of MBC populations^62^. Furthermore, a study conducted in Brazil suggests that one or more boosters of YFV 17DD following primary vaccination helped to restore the levels of T and MBC responses and prevented the progressive decline in NAb titers^63^.****

### ZIKV

ZIKV is a re-emerging flavivirus that has caused outbreaks in recent years in the Americas and Caribbean^64–66^. The virus can be transmitted by mosquito bites or by sexual contact^67–69^. In addition, the virus has been associated with severe neurological diseases, such as the autoimmune disorder Guillain-Barré syndrome in adults and congenital Zika syndrome in fetuses and infants^70–72^.

The ZIKV E protein is responsible for viral entry into host cells and represents a major target for NAbs^73^. NAbs targeting the EDIII protein have also been shown to protect mice against lethal ZIKV infection^9^. One early study of plasma of ZIKV patients from a 2016 outbreak in Singapore suggests that the majority of patients had robust ZIKV-specific humoral responses. Anti-ZIKV IgM was detected as early as 2 days post illness onset and peaked during the 10 to 14 day period post illness onset before decreasing at the 3 month to 1 year recovery phase. Anti-ZIKV IgG peaked during the 10 to 14 day period, persisted at high levels for 5 to 6 months, and was still detectable 1 year post infection^3^.

B cells induced in DENV patients are known to share epitopes between DENV serotypes and other flaviviruses along with epitopes unique to each serotype. A multifunction FluoroSpot assay using fluorescently labeled DENV and ZIKV was utilized to simultaneously detect DENV serotype-specific, ZIKV-specific, DENV serotype cross-reactive, and DENV/ZIKV cross-reactive antibodies secreted by individual MBCs following vaccination or natural infection. Although ZIKV is closely related to DENV, minimal DENV and ZIKV cross-reactive MBCs were detected when samples from ZIKV-immune patients were analyzed^40,74^. Antibodies specific for ZIKV EDI/II were cross-reactive, but not neutralizing DENV, which resulted in lethally enhanced DENV disease in mice, though this has not been observed in humans^9,75–77^. Andrade *et al*. characterized ZIKV-specific MBCs and serum binding and NAb responses to ZIKV at both 2 weeks and 8 months after infection in 31 pediatric patients with (a) no, (b) one, or (c) more than one related prior DENV infection. They found that ZIKV induced robust type-specific MBC responses. ZIKV-specific antibodies contributed to anti-ZIKV serum neutralizing activity during the late convalescent stage. Furthermore, prior immunity to DENV only modestly shaped the breadth and magnitude of the MBC responses^74^. Similar results were confirmed in another study in which single B cell cloning and large-scale antibody isolation were used to characterize ZIKV-induced B cell responses in three DENV-experienced donors^78^.

Several studies have characterized human B cells in ZIKV/DENV immune donors. Robust plasmablast populations were elicited in DENV experienced donors during acute ZIKV infection, whereas the magnitude was reduced in DENV immune donors. The plasmablast response during acute ZIKV infection peaked at day 7 post presentation of symptoms and became undetectable by day 15^79^. The plasmablasts from the DENV/ZIKV immune subject were expanded with a high level of somatic hypermutation (SHM) similar to secondary DENV infection, and a similar effect was also observed in MBCs and plasmablasts generated by influenza vaccination^80^. In contrast, the plasmablast in the DENV-naïve/ZIKV-immune subject was characterized by low frequency, limited clonal expansion, and low SHM^78^. While plasmablast-derived mAbs in DENV exposure donors showed ADE effects, mAbs from ZIKV-infected patients without prior DENV exposure showed less cross-reactivity to DENV serotypes^81^. At 5 months post infection, MBCs included a mixture of broadly cross-reactive, poorly neutralizing, and *de novo* generated antibodies that were ZIKV specific and potently neutralizing^78^. Overall, although prior DENV infection has been associated with ADE following ZIKV infection, the complexity of it suggests that further investigation is needed.

## Summary and future perspectives

Humoral immunity, including MBCs and antibody responses, are important for the control of flaviviral infection and host protection. During primary flaviviral infection, there is a rapid expansion of antibody-secreting plasmablasts and induction of virus-specific MBCs. Upon secondary flavivirus infection, MBCs are mostly characterized as highly cross-reactive and secrete antibodies with higher avidity to genetically related flaviviruses.

Co-circulation and epidemiological overlapping of more than one flavivirus in endemic areas has often resulted in the generation of massive cross-reactive MBCs and antibody responses, which are likely to have a strong impact on the development of an effective vaccine and accurate diagnosis of flavivirus infections. MBCs induced during primary DENV infection are known to share epitopes between DENV serotypes and other flaviviruses along with epitopes unique to each serotype. Plasmablast-derived mAbs in DENV patients are broadly cross-reactive against DENV 1–4 serotypes and contribute to the ADE effects^31,82^. There are minimal DENV and ZIKV cross-reactive MBCs, though the two viruses are closely related. Despite the fact that significant work has been conducted on the understanding of the specificity and cross-reactivity of antibody responses to DENV infection, little is known about the role of MBCs and their derived antibodies in other flavivirus infections. Innate immune signaling pathways and inflammatory cytokines are involved in the regulation of MBC development. However, the underlying immune mechanisms of MBC development during flavivirus infection and vaccination remain unclear and will be the focus of future investigation.
